# The role of 3D printing in anatomy education and surgical training: A narrative review

**DOI:** 10.15694/mep.2017.000092

**Published:** 2017-06-06

**Authors:** Ka Hou Christien Li, Christopher Kui, Elgin Kah Meng Lee, Cheuk Sang Ho, Sunny Hei Wong, William Wu, Wing Tak Wong, Jessika Voll, Guangping Li, Tong Liu, Bryan Yan, Jessica Chan, Gary Tse, Iain D. Keenan

**Affiliations:** 1 Newcastle University; 2 Chinese University of Hong Kong; 3 Newcastle upon Tyne Northern Deanery; 4 Tianjin Medical University; 5 University of Oxford

**Keywords:** 3D-printing, Anatomy, Surgery, Undergraduate Education, Postgraduate Training

## Abstract

This article was migrated. The article was marked as recommended.

Recent expansions in the development and availability of three-dimensional printing (3Dp) have led to the uptake of this valuable and effective technology within the modern context of medical education. It is proposed that 3Dp is entirely appropriate for the creation of anatomical models for purposes of teaching and training due to the ability of this technology to produce accurate 3D physical representations based on a processed data set acquired from sources including magnetic resonance imaging (MRI) and computed tomography (CT). When investigating the currently available educational research with respect to 3Dp, it is important that the best evidence supporting the practical and theoretical benefits of this technology in teaching and training can be identified, while any obstacles to the effective implementation of 3Dp can also be determined. Here, literature describing recent primary research with respect to the capability and utility of 3Dp in anatomy and surgery have been explored in a narrative review. The impact on resources of implementing this technology within medical education have also been investigated. In order to emphasise wider applications in medicine, the role of 3Dp in medical practice and research have also been examined. To identify recent literature appropriate for this review published up to March 2017, suitable search terms were determined and applied using PubMed and results were judged against an established checklist. The research identified was then allocated with respect to the agreed topic areas of anatomy education, surgical training, medical usage and medical research. A student partnership approach was utilised for this review and the focus of the work was driven by undergraduate students in collaboration with anatomy and medical educators. Preliminary findings from this narrative review support the implementation of 3Dp in anatomy education and surgical training as a supplement to traditional learning approaches.

## Introduction

Anatomy, in essence, is a discipline concerned with the three-dimensional structure of living things. The study of human anatomy has historically been the cornerstone of medicine, extending from the earliest examinations of sacrificial victims to the sophisticated analyses undertaken today. From the time of Galen in the 2nd century AD, the study of gross anatomy and anatomical concepts have been incorporated into medical curricula. The importance of anatomy also extends to those in surgical specialties due to the direct relevance of the discipline to clinical practice ( Arraez-Aybar et al., 2010; Fredricks & Wegner, 2003; Lindemann, 2010; Martin et al., 2014; Smith and Mathias, 2011). Traditional or standard anatomy teaching methods primarily consist of didactic lecture-based teaching, e-learning and small group teaching involving cadaveric dissection, prosection and anatomical models. Approaches concerning clinically relevant surface and radiological anatomy are also often included in teaching ( Kerby et al., 2011), while simulation tools such as ultrasound, arthroscopy and technology-enhanced learning (TEL) are also used ( Griksaitis et al., 2012; Hammoudi et al., 2013; Jurjus et al., 2014; Knobe et al., 2012).

Evaluation of the effectiveness of anatomy teaching is multi-factorial, as established by multiple studies. Quantitative measures include short-term knowledge acquisition and long-term retention, in addtion to student confidence and satisfaction ( Brown et al.2012; Chen et al., 2010; Chinnah et al., 2011; Preece et al., 2013). In traditional dissection, students commonly work in small groups in order to facilitate multi-sensory understanding of anatomical relationships in three dimensions ( Older, 2004; Singh, 2013). The subtle introduction of the concept of humanistic care and respect is integral to this experience ( Rizzolo, 2002). Animal specimens and 3D laparoscopic dissection models, in some cases, are utilised instead of the traditionally prepared cadaver as a result of increased cost ( Musumeci et al., 2014; ten Brinke et al., 2014). Regardless of whether cadaveric models or alternative animal dissected models are used, a positive experimental outcome has been observed when compared to normal didactic teaching sessions incorporating the use of plastic models ( Musumeci et al., 2014; ten Brinke et al., 2014). However, improvements with alternative dissected models were limited only to short-term knowledge acquisition, and were not detected at 2 week follow-up. It is also worth noting that the former approach with dissected models also increased teaching duration by 3 hours, which may have resulted in the improved short-term knowledge acquisition recorded ( ten Brinke et al., 2014). When taken together with the increasing costs of traditional methods, particularly dissection ( Bergman et al., 2014; Collins, 2008; Drake et al, 2009; Turney, 2007), the decline in anatomy teaching in undergraduate programmes, and the rise of technology now utilised in medical education, this has led to research and development of new and innovative ways of teaching anatomy ( Collins, 2008; Lewis, 2003; Tam, et al., 2009; Turney, 2007; Yammine and Violato, 2015). Such approaches include computer-assisted instruction (CAI) and computer-assisted learning (CAL) as modern alternatives ( McLachlan and Patten, 2006; Older, 2004; Benly, 2014; Papa & Vaccarezza, 2013; Sugand et al., 2010; Turney, 2007). Three-dimensional printing (3Dp) has also emerged as a novel teaching tool for anatomy ( AbouHashem et al., 2015; Adams et al., 2015; Lim, et al., 2016; O’Reilly et al., 2016), with its effectiveness being reflected in the findings of a recent randomised controlled trial (RCT) conducted in China ( Li et al., 2015).

3Dp, also known as additive manufacturing (AM), refers to the processes used to create a three-dimensional object. 3Dp models can be created with computer-aided design (CAD) package, using a 3D scanner, or by a plain digital camera and photogrammetry software. Since design verification occurs before the printing process, 3Dp models are highly accurate. The optimal method of teaching anatomy is still widely debated, with each medical school taking a different approach. Despite dissection being an effective method of meeting learning outcomes, this approach is unlikely to address all aspects of the curriculum, as is the case for any other single teaching modality. As such, a complementary multi-modal approach is recommended for modern medical school anatomy courses ( Kerby et al., 2011).

This review seeks to evaluate the potential role and effectiveness of 3Dp, not as an replacement for established approaches, but rather as a cost-effective, efficient and complementary modality to aid in modern medical education in the context of anatomy teaching and specialty training. Aspects of the use of 3Dp in medical practice and impacts on medical research are included to illustrate the wider roles of 3Dp in medicine.

## Methods

A PubMed search for studies that primarily concerned the application of 3Dp in medical education and training was conducted. In order to do so, the following search string was employed: [“3D printing” AND (“medical education” OR “anatomical models” OR “anatomy education” OR “rapid prototyping” OR “medical teaching” OR “imaging techniques” OR “anatomical sciences” OR “ultrasound simulation” OR “residency training” OR “radiology education”)]. The search period was determined as beginning from the earliest available publications in the database (1965 for PubMed) through to 31st March 2017. No language restrictions were included.

The search results were subsequently transferred to Microsoft Excel (Microsoft Corp., Redmond WA) and all potentially relevant reports, which were consulted with the Preferred Reporting Items for Systematic Reviews and Meta-analyses (PRISMA) checklist, were retrieved as complete manuscripts and assessed for compliance with relevance to the four core principles of this review: (1) Undergraduate medical education; (2) Postgraduate training; (3) Medical usage; (4) Medical research. Three authors (CL, CK and EL) independently reviewed each publication and report and findings were agreed upon by consensus with input from two senior researchers (GT and IK).

Extracted data elements of RCTs included in the review consisted of study design; follow-up duration; endpoint(s); and characteristics of the population including sample size, gender, and age. This was in accordance with PICO (Patient, problem or population; intervention; comparison control or comparator; outcome eligibility) criteria. The endpoints of the study included short-term knowledge acquisition, long-term retention, student confidence levels in their knowledge, student satisfaction and/or cost-effectiveness.

## Undergraduate Anatomy Education

### 3D printed models for gross anatomy and pathology education

The utility of 3Dp models within medical education is evident in that data can be acquired from a large range of sources to produce an endless variety of models. These models in turn can be printed in a multitude of materials, with the added potential for customisation after production. With sufficient funding, a medical school could produce learning models suitable for use in a range of fields which extend from and beyond anatomy. One of the benefits of 3Dp is the reintroduction of anatomical variation in the advent of declining cadaveric dissections ( Fasel et al., 2016; Moore et al., 2017; Nieder et al. 2004; O’Reilly et al., 2016). Currently utilised plastic anatomical models and atlases are idealised and do not include the anatomical variations found during cadaveric dissection. Previously proposed solutions include the digitisation of such variations and storing them virtually as 3D images ( Moore et al., 2017; Nieder et al., 2004). A tactile experience could be incorporated whereby 3Dp models could be constructed from existing 3D images, providing students and lecturers with the ability to handle and manipulate these variations. In one instance, students were permitted to undergo cadaveric dissection, scan the cadavers using computed tomography and produce 3Dp models. This generated anatomical variations, such as a left coronary artery trifurcation. Such variations are preserved and can be experienced by future generations of students ( Fasel et al., 2016).

It is possible that 3Dp may possess qualities that may provide students with a gross anatomy learning experience that includes aspects equivalent to, if not more effective than, that of cadaveric dissection. A recent exploration as to whether anatomical teaching resources could be produced at sufficient resolution to provide metrics of high concordance with the original specimen has been conducted ( McMenamin et al., 2014). A high-quality upper-limb prosection was scanned using CT imaging and re-created as a colour-coded 3Dp model, evaluation of which positive resulted in positive findings. Aside from possessing aesthetically pleasing visuals, the limb was printed accurately with terminal neuro-vasculature easily identifiable. The model was reproduced in several sizes so a larger model could be kept permanently at the anatomy laboratory, whereas smaller ones could be more easily manipulated and could be taken outside of the classroom for self-directed learning. It is also important to note that 3Dp models could mitigate health and ethical concerns surrounding biological specimens, conferring the advantage of extending anatomy learning opportunities outside of the anatomy laboratory and into a clinical environment. In this way, clinicopathological correlation could be provided at multidisciplinary team meetings ( Mahmoud & Bennett, 2015).

Pathology courses stand to benefit greatly from the introduction of 3Dp models through provision of an extra dimension to this traditionally didactic discipline. Data can be sourced from a variety of locations, such as healthy volunteers, patients and cadavers. Digital libraries shared between institutions and anatomical museums could also be used to gain access to rare and declining diseases ( AbouHashem et al., 2015; Moore et al., 2017; Nieder et al., 2004). However, surgical specialties would most likely become a primary source of educational models, as those created for pre-operative planning can be re-used in the classroom ( Bizzotto et al., 2015). A variety of 3D-printed models have been produced by numerous specialties ( Marro et al., 2016), ranging from comminuted distal tibial fractures ( Chung et al., 2015) to pulmonary vasculature ( Kurenov et al., 2015) and the renal system ( Powers et al., 2016).

Many medical students will observe tumours only in a non-stimulating setting such as a textbook, presentation slide, CT slice, or rarely, in a preserved sample. A 3Dp model however, can provide a multi-sensory learning experience. Highly accurate bone ( AbouHashem et al., 2015), kidney ( Knoedler et al., 2015; Powers et al., 2016), lung, liver and breast tumours ( Jones et al, 2016) have been printed. Such models are complemented by contrast CT, which can present surrounding vasculature and ‘negative’ structures such as air sinuses. (Kong et al.; Kurenov et al., 2015; Lim et al., 2016; Maragiannis et al., 2015; McMenamin, et al., 2014; O’Reilly et al., 2016). These sophisticated models can also be digitally painted to enhance student learning, providing a learning advantage over monochromatic models ( Ejaz et al., 2014). To provide a more tactile experience, there is potential for constructing soft-tissue and tumours from different materials. For example, a ‘soft breast’ with ‘rigid mass’ model constructed in this manner could be used to teach the clinical skill of palpation ( Jones et al., 2016). 3D-ultrasound (3DUS) technology can increase the scope of 3Dp to additional medical specialties. In the case of challenging topics such as embryology and development where dynamic changes occur in 3D, ultrasound can be used in conjunction with MRI to construct an accurate image of a fetus within the uterus ( Werner et al., 2010). In this manner, normal and abnormal development can be captured, providing students with a 3D visual aid while reducing ethical concerns. This method can also be used to produce physical fetal models for pathologies ranging from cleft lip to achondroplasia dwarfism ( Werner et al., 2010).

At greater expense both financially and temporally, more educationally innovative models have been designed. They include colour coding ( Chen et al., 2017; Kong et al.; McMenamin et al., 2014; O’Reilly et al., 2016), aneurysms that can be ‘opened’ to reveal internal structure ( Jones et al., 2016), multi-material printing to represent calcified aortic valves ( Maragiannis et al., 2015) and the addition of partitions to illustrate hepatic segments ( Kong et al., 2016). In a recent study, a 3D lower limb model consisting of the bones and calf muscles was printed ( O’Reilly et al., 2016). The bones of the leg and foot were printed separately and had magnets incorporated post-printing, allowing them to be attached and detached. Tendons were printed with iron filaments and incorporated into silicon calf muscles, thus providing a contrasting texture and allowing detachment from the bone portions of the model. A similar model could potentially be used to teach complex areas with multiple layers of muscles.

### Experimental research

Findings from randomised controlled trials can be used to illustrate the practical impact of 3Dp models in anatomy education. However, despite well-established usage by many medical schools, particularly in Australia and the United States, there are limited quantitative comparisons between 3Dp models and other methods of anatomy teaching. Traditionally dominated by the use of cadaveric dissections and anatomical atlases ( Drake and Pawlina 2014; Elizondo-Omaña et al., 2005), multiple logistical, financial and ethical issues have resulted in the declining use of cadaveric dissection ( AbouHashem et al., 2015; McMenamin et al., 2014; Vaccarezza and Papa, 2015). Various tools including prosections, plastic ‘idealistic’ models, plastinated specimens, body painting and 3D digital images were introduced to compensate but have appeared to be most effective when used in combination with cadaveric dissection ( Drake and Pawlina, 2014; Elizondo-Omaña et al., 2005; McMenamin et al., 2014; Vaccarezza and Papa, 2015), with each tool having particular advantages and disadvantages. The addition of 3Dp to the modern array of educational approaches in anatomy is promising and may eventually replace other adjuncts. Though cadaveric dissection may never disappear entirely, 3Dp models can provide a strong alternative where appropriate. A 3Dp upper limb is durable and accurate, comparable to that of plastinated specimen ( McMenamin et al., 2014). While both 3Dp and plastination do not provide the experience of real tissue, 3Dp is an evolving technology and producing soft, hyper-realistic 3Dp limbs, organs, bones and other items may eventually become financially as well as educationally feasible.

3D virtual (3Dv) models have been used in an educational setting for a number of years. Within this context, 3Dv refers to the use of modern imaging modalities to convey the impression of a 3D space. When compared to 2D representations of human structures, the use of 3Dv results in improved outcomes in terms of understanding spatially demanding anatomical knowledge ( Hoyek et al., 2014) and student satisfaction ( Beermann et al., 2010; Hoyek et al., 2014). However, improved outcomes only seem to occur with respect to complex anatomy ( Beermann et al., 2010; Hoyek et al., 2014). Though an excellent teaching tool, 3Dv lack the physical experience of a cadaver, but it may be possible to overcome this barrier if virtual models can be converted into 3Dp models. A randomised-controlled trial was carried out to compare the effectiveness of an anatomical atlas, a coloured 3Dv model and a coloured 3Dp model when teaching hepatic segment anatomy ( Kong et al., 2016). Having received a pre-exposure test and short introductory lecture, first year medical students spent an hour with one of three tools before being assessed on theory and labelling of structures. The two 3D groups achieved a statistically significant higher post-exposure score than the ‘2D’ anatomical atlas group, both in the immediate assessment and a ‘surprise’ test 5-days later. Another study echoed this finding ( Li et al., 2015). 2D CT images, monochromatic 3Dv and monochromatic 3Dp models were used to deliver teaching with respect to cervical and thoracic spinal anatomy and fractures. In a 10-mark examination, the 3Dv and 3Dp groups averaged a post-exposure score higher than the CT group. These studies demonstrate the effectiveness of 3D learning methods over 2D methods and may suggest improved long-term knowledge retention. However, as no comparison to cadaveric specimens have been made, these studies primarily promote usage of 3D-printed models for home or classroom-based learning, outside of the anatomical laboratory.

Findings indicate that 3Dp models could be effective to an equivalent extent to cadaveric prosections for teaching anatomy. Upon conducting a formative test following a 1-hour practical session comparing a cadaveric model and the 3Dp lower-limb model, no statistically significant difference was found in the average score (45.5% vs 50%, p = 0.46) ( O’Reilly et al., 2016). Similarly, no statistically significant difference in theory test scores were reported when comparing an anatomical atlas, painted cadaveric skull and digitally painted 3Dp model (p = 0.669) for the teaching of skull anatomy ( Chen et al., 2017). However, in a 30-mark structure recognition test, first year medical students in the 3Dp group scored higher than both the cadaveric and atlas groups (16.5, 14, 14.5, p = 0.049). A significant positive effect to using digitally painted 3Dp models for delivering teaching concerning the heart, great vessels and coronary vasculature has been reported ( Lim et al., 2016). Another group used cadaveric specimens, with the final group using a combination of both approaches. The 3Dp-only group scored the highest in the post-exposure test, a statistically significant difference compared to the other group scores (p = 0.012). There was no significant difference in scores between the cadaveric-only and combined groups.

A consistently elicited result relates to that of student perception and satisfaction. 3D models in any form are preferred over 2D images ( Hoyek et al., 2014; Li et al., 2015). This impact is greater in 3Dp models compared to 3Dv models ( Li et al., 2015), with students reporting greater pleasure, personal learning effect and confidence in explaining to peers. 3Dp and cadaveric skulls were allocated strongly positive ratings by over 85% of the students in the categories of enjoyment, intention to use, attitude and learning efficiency ( Chen et al., 2017). Qualitative assessments of 3Dp projects in anatomical education have also received strong positive feedback ( AbouHashem et al., 2015; Fasel et al., 2016; Jones et al., 2016; Knoedler et al., 2015).

Taken together, these studies demonstrate that 3Dp models can be effective teaching tools. It is therefore recommended that where possible, 3Dp models should be utilised for delivering anatomy teaching in circumstances where only anatomical atlases or other forms of 2D images have been previously used. Though not necessarily more effective for delivering information than using 3Dv images, 3Dp models can provide an important tactile experience and are preferred by students ( Marconi et al., 2017). An important gender disparity has been observed with respect to 3Dv images ( Beermann et al., 2010; Zhenzhu Li et al., 2015), with females shown to learn less effectively. This difference has not been observed with 3Dp models ( Chen et al., 2017; Li et al., 2015; Lim et al., 2016). When compared to cadaveric prosections, results consistently illustrate 3Dp models are equally beneficial teaching tools and may be more beneficial in teaching practical anatomy ( Chen et al., 2017). Cadaveric specimen groups performed below expectations, with explanations including the presence of damaged bone that had been digitally repaired for the 3Dp model, a lack of colour heart prosections, and a well-documented discomfort of first-year students towards cadaveric specimen ( Aung and Tin, 2012).

### Financial and logistical considerations

Despite the advantages of 3Dp in medical education, there have been concerns with respect to the cost and time needed to introduce a 3D printer and regularly produce models. However, as the technology has developed, there has been a general decrease in costs, with prices of an equivalent 3Dp model comparable to or possibly less expensive than those of ‘idealistic’ plastic models ( Balestrini and Campo-Celaya, 2016). If recent increases in the popularity and utility of 3Dp continues, costs may fall and further model templates will likely become available for purchase. This may lead to institutions beginning to regularly produce their own models for teaching and training purposes. Costs can vary greatly depending on the situation and intention of the user: a medical student could use a $200 (USD) desktop printer, free software and publicly available designs to construct simple and durable plastic models for self-study ( McMenamin et al., 2014). However, a medical school planning to generate sophisticated educational tools could spend significant amounts for the initial set-up costs. In order to generate a 3D-printed model of the upper limb prosection, $65,000 was required to purchase a powder printer and over $10,000 for CT imaging, computers and software ( McMenamin et al., 2014). However, with the initial set-up complete, the 3Dp upper-limb model had a final material cost of $300-350, versus $14,000 plastinated upper limbs and $1800 plastic models. Comparably, acquisition of a cadaver was estimated at $8500, not including dissection fees ( McMenamin et al., 2014). The relatively low cost of 3Dp is best highlighted by simple models. Life-size 3Dp plastic skulls and brains have a material cost of below $15 ( Chen et al., 2017; Naftulin et al., 2015), with a spinal model containing multiple vertebrae and fractures costing $20 (Zhenzhu Li et al., 2015). The costs of printing softer, silicon models can be greater depending on the functionality required, but using 3Dp plastic moulds and injecting medical-grade silicon can reduce the costs involved significantly ( Chung et al., 2014; He et al., 2014). This method has been used to produce silicon ear prosthetics for $30, and further savings can be made when moulds can be re-used. However, adding extra features and creating patient-specific 3Dp models can increase costs. While digital painting is of relatively low cost, designing and producing partially transparent, multi-texture models or modular features can be more expensive. Creating multiple complex models containing patient pathologies, such as a 3Dp translucent model of an aneurysm that could be taken apart to study internal anatomy, resulted in a costs of $2700 for processing, design and production for self-study ( Jones et al., 2016).

Another concern are the time costs necessary for production of 3Dp models. This varies depending on the size of a model and the type of printer. A powder-based 3D printer takes three hours to produce a life-size hand prosection replica, complete with colour and neuro-vasculature ( McMenamin et al., 2014), whilst the complex aneurysm model described above took seven days to print using an inkjet 3D printer ( Jones et al., 2016). Fortunately, the printing process is not labour-intensive and requires minimal supervision. Segmentation, on the other hand, an important part of processing 2D images to ensure accurate and high quality prints and requires intensive manual labour. For example, medical students segmenting for the first time took up to 40 hours for the small and large intestines ( Fasel et al., 2016), although this time is likely to be reduced with training. It is proposed that 3Dp models should be used as a major educational tool for undergraduate anatomy learning, with evidence suggesting that this approach also has the potential to enhance radiology and pathology teaching ( Knoedler et al., 2015; Li et al., 2015) by providing case-based discussions with interactive visual aids. It is proposed that medical students should still be exposed to cadaveric specimens ( Elizondo-Omaña et al., 2005), with 3Dp models serving to supplement standard methods and to substitute in place of poor quality or damaged prosections. Further research may support the regular usage of 3Dp models within anatomy curricula ( Sullivan, 2011; Ware and Hamel, 2011), with direct comparisons to ‘idealistic’ plastic models, plastinated specimens and a cadaveric dissection session necessary.

### Summary: 3D printing in undegraduate anatomy education

It is proposed that 3Dp models should be used as a major educational tools for undergraduate anatomy learning, with evidence suggesting that this approach also has the potential to enhance radiology and pathology teaching ( Knoedler et al., 2015; Zhenzhu Li et al., 2015) by providing case-based discussions with interactive visual aids. It is proposed that medical students should still be exposed to cadaveric specimens ( Elizondo-Omaña et al., 2005), with 3Dp models serving to supplement standard methods and to substitute in place of poor quality or damaged prosections. Further research may support the regular usage of 3Dp models within anatomy curricula ( Sullivan, 2011; Ware and Hamel, 2011), with direct comparisons to ‘idealistic’ plastic models, plastinated specimens and a cadaveric dissection session necessary.

## Postgraduate Surgical Training

### 3D-printing in pre-operative surgical training

In addition to the potential of 3Dp for the delivery of undergraduate teaching with respect to both normal and abnormal anatomy, many investigators have acknowledged a possible role for 3Dp in the training of postgraduates in clinical procedures. In the field of surgery, the potential for 3Dp models has been explored for advanced simulation training that could be patient-specific, incorporate pathology and can share compatibility with surgical implants ( Kurenov et al., 2015; Maragiannis et al., 2015; Narayanan et al., 2015). Physical models for more advanced clinical skills such as femoral vessel access that is compatible with a pulse generator and can be detected using Doppler ultrasound ( O’Reilly et al., 2016). Researchers have also developed the physical properties of 3Dp materials when creating a 3Dp eye model that can potentially be used for funduscopic training ( Xie et al., 2014).

The use of 3Dp models as pre-operative training tools provides multi-planar visualisation of anatomy and its relevant pathology. Additionally, 3D printed models can be produced using alternative materials to mimic specific types of tissue such as cardiac, tracheal, vasculature and bones ( Costello et al., 2015; Javan et al., 2016; Jehad et al., 2016; Otton et al., 2015; Ryan et al., 2016). When integrated with patient-specific models, 3Dp displays clear advantages over traditional cadaveric models since it allows surgeons to undergo a realistic simulation of the individualized procedure required before the actual surgery (Figen Govsa et al., 2017; Shin et al 2016). Such training clearly has many benefits including a reduction in operation time ( Matthew et al., 2016) and the ability to predict intra-operative complications ( Matthew et al., 2013). They also assist in pre-operative planning ( Riesenkampff et al., 2009; Ralf Sodian et al., 2008). Therefore, when 3D models are used in tandem with other resources such as video recording and feedback, this approach provides optimal training across many fields, both surgical and medical ( Costello et al., 2015).

Simulations offer the capability to create realistic medical scenarios for surgical training procedures and provide space for errors without any risks to patients ( Dimmick et al., 2007; Ganju et al., 2013; Issenberg and Scalese, 2008; Stan and Ingrid, 2012), especially in particular challenging procedures ( Narayanan et al., 2015). Due to the importance of surgical skill development, the Accreditation Council for Graduate Medical Education in the United States now requires simulation-based training for general surgery residencies ( Stan and Ingrid, 2012) and the three basic approaches to this training involve human and animal cadaveric specimens, digital and virtual reality, and synthetic physical 3D models ( Ryan et al, 2016). Since the inception of body bequest programmes, donated cadaveric specimens have become a valuable resource used in teaching anatomy to both medical students and postgraduate trainees. However, since the 1970s, the numbers of such specimens have declined ( Jonas, 2017). That, combined with other factors such as the high cost of maintaining cadavers and the various ethical concerns associated with the use of human bodies, raises an urgent need to find and utilise alternative resources ( Shui et al., 2017). With respect to postgraduate training, donor specimens with specific lesions or pathologies are particularly limited ( Ryan et al., 2016). Further problems can arise if animal specimens are used due to the inevitable anatomical differences ( Jehad et al., 2016).

In light of recent advancements in imaging, an alternative simulation tool for surgical training is virtual reality (VR) or 3D digital imaging. However, clinicians including physicians, nurses and ancillary care providers are still more likely to consider 3Dp models to be more helpful than 2D images when visualizing complex cases (Laura et al., 2016). Additionally, VR and 3D digital imaging do not always demonstrate spatial relationships efficiently ( Shui et al., 2017) and may not always provide an accurate depiction of the anatomy ( Jonas, 2017) or to a satisfactory level ( Rahal et al., 2014). Furthermore, these approaches do not offer trainees the chance to perform the actual procedure and hence gain the higher cognitive, psychomotor and affective skills required fpr translation into technical skills ( Khan et al., 2011).

3Dp models, derived from a variety of imaging modalities including CT and CT Angiography (CT-A), Cone Beam CT, MRI Time-of-flight, MR angiography and even echocardiography ( Jonas, 2017; Marro et al., 2016; Shui et al., 2017) provide a further approach to developing surgical simulations. Such 3Dp models have several advantages over cadaveric material, in that they are cheap and quick to produce, safe, reusable and can be scaled to any size ( Da Cruz and Francis, 2015; Govsa et al., 2017; Jehad et al., 2016; Mowry et al., 2015). Additionally, 3Dp models do not present the same logistic challenges as cadaveric specimens as they are non-biohazardous so do not require special laboratories or instruments and are relatively simple to dispose of ( Jehad et al., 2016). Compared to digital representations, 3Dp models demonstrate spatial relationships more effectively and are therefore useful in training and education ( Govsa et al., 2017; Laura et al., 2016; Shui et al., 2017).

Not only can 3Dp circumvent some problems relating to usage of cadaveric material and digital models, it can also enhance certain aspects of surgical training. 3Dp can improve the learning of surgical anatomy and specific diseases with key pathological features, can develop both basic handling and microdexterity with respect to particular procedures, and can improve and reconcile prior knowledge obtained from more traditional learning approaches ( Malik et al., 2015; Ujiki & Zhao, 2011). These skills can become even more important in certain fields. For example, pre-operative investigations may at times lack the accuracy needed to demonstrate the actual anatomy in congenital surgical procedures ( Jonas, 2017) and serious complications may arise intra- and post-operatively due to limited experience or training during high risk surgery ( Govsa et al., 2017). Further to pre-operative training, the benefits of 3Dp models are also evident in intra-operative guidance and post-operative management ( Kong et al., 2016; Shin et al 2016).

### 3D-printing in intra-operative guidance

In some cases, the implementation of 3Dp complements the lack of surgical practice opportunities at residency training level, as seen in open cerebral artery aneurysm surgeries ( Lai and Morgan, 2012; Ryan et al., 2016). Despite the introduction of endovascular interventions, depending on the location, size and lesion morphology, open aneurysm surgery may remain as the preferred approach ( Burns and Brown, 2008; Li et al., 2012; Ryttlefors et al.,, 2008). With advancement in 3Dp technology, it is now possible to create a very realistic model of cerebral artery aneurysms, which consist of the skull, brain and the vasculature itself ( Ryan et al., 2016). These three components, when used in combination, provide a very life-like depiction of the actual procedure of clipping aneurysms. For example, the skull model responds well to craniotomy, the brain model is of the appropriate turgor and recoil to recreate the surgical experience, and last but not least, the hollow nature of the 3Dp blood vessel aneurysms are collapsible when a clip is applied, mimicking the real procedure. Additionally, the combined model demonstrated vasculature relationships, branching, surgical landmarks and the realistic, limited field of vision that can be obtained after a standard craniotomy. Furthermore, due to the segmental nature of the model, it is possible to insert specific aneurysms depending on patient-specific pathology or even just to suit particular teaching objectives without having to replace the entire model. That also accommodates the replacement of individual worn out parts at lower cost ( Ryan et al., 2016).

The benefits of 3Dp models are clearly seen in patients with challenging anatomy. Under head and neck surgery, the cervical portion of the internal carotid artery (ICA) has been established as having a straight course to the base of the cranium without branching ( Cappabianca et al., 2016; Lien et al., 2014). However, variation in the anatomical course of the ICA has been reported in up to 40% of the population, even amongst those who have no history of relevant long term conditions, such as cardiovascular disease or diabetes ( Cvetko, 2014; Katsuno et al., 2014; Omer et al, 2010). These geometrical anomalies include highly angulated, conical or short necks, as well as narrowed lumens and tortuous or coiled arteries ( Govsa et al., 2017), the latter being indiscernible pre-operatively ( Yu et al., 2016). This presents a problem in head and neck surgery, where relatively routine procedures such as tonsillectomy, adenoidectomy and drainage of peritonsillar abscess may result in massive life-threatening haemorrhage, both intra- and post-operatively, especially when performed by less experienced surgeons ( Cvetko, 2014; Lien et al., 2014; Yu et al., 2016). There is also an associated increased risk of stroke due to the tortuosity ( Govsa et al., 2017).

While CT-A can detect coiling of the ICA preoperatively, it is unable to provide the level of detail of a 3Dp model ( Rahal et al., 2014). Therefore, for each patient with challenging carotid anatomy as detected by CT-A, producing a life-size 3Dp model offers surgeons a tool which can be used to assist in both training and pre-operative planning, such as accurate selection of stent-graft type and potential intra-operative adjuncts ( Govsa et al., 2017). Another study compared the effectiveness of 3Dp models versus porcine models in the training of foreign body removal via rigid bronchoscopy. It was concluded that the 3D models were a valid alternative to animal models in terms of trainee satisfaction with regards to various aspects such as anatomical accuracy, realism, level of difficulty and translation into better outcomes when actually performing the procedures. It was noted that 3Dp models had several advantages over the porcine models. They were more anatomically correct, since they were based on a template of an actual patient, and it was possible to scale the models to cater to different age groups by adjusting the airway dimensions as necessary and by altering the concentrations of the polymer, based on a certain formula ( Gent, 1958), to mimic varying tracheal elasticity ( Jehad et al., 2016). Instead of printing new models, a study on CT-guided spinal pain management, noted that further customisations could be made to the models to include various pathologies based on real patient data, which would then allow trainees to practice procedures beyond the scope of pain management, for example, vertebroplasties, biopsies, aspiration of synovial cysts and even blood patching in the case of intracranial hypotension ( Javan, et al., 2016; Kranz et al., 2011).

Having considered how 3Dp models are able to mimic specific pathology hence assist in training, it is also important to address the beneficial but less common usage of replicating normal anatomy, particularly with respect to orthopaedic surgery. In the case of distal tibial fractures, repair is commonly performed using anatomically contoured locking plates which can be obtained from various manufacturers ( Ozkaya et al., 2009; Song et al., 2013). However, such plates are generic in the sense that while there is range from which a selection can be made, they are designed based on the measurements and shape of an average human. Therefore, occasionally, a mismatch might arise in patients with a tibia that is considerably smaller or larger, or in patients with tibial deformities ( Liang et al., 2014; Oh et al., 2010; Song et al., 2013). In such patients, using the 3Dp model of normal anatomy can assist in the repair of the abnormal part. For example, a model of an intact left distal tibia would allow surgeons to select the best-fit plate to fix a fractured right distal tibia, since the mirror image is likely to be representative of the damaged bone pre-injury ( Kook Jin et al., 2015).

The pre-operative and intra-operative use of a 3Dp model may also reduce the experiential gap between generations of surgeons. More successful pre-operative planning in trainees when using a 3Dp model has been demonstrated compared to virtual 3D renditions ( Zheng, et al., 2016) and the ability to highlight dangerous anatomical variations, such as those of the extracranial internal carotid artery when operating at the skull base has been described ( Govsa et al., 2017). When an operation is not successful, 3Dp models can be created post-mortem in order to analyse whether the correct interventional choices were made. A patient’s ‘porcelain aorta’ has been printed after a transcatheter aortic valve implantation (TAVI) procedure resulted in an ischaemic event and death, and allowed identification of abnormal anatomy that was incompatible with the normal valve deployment site ( Schmauss et al., 2015).

### 3D printing in post-operative care

An example of the use of 3Dp models in post-operative care concerns congenital cardiac surgery patients (Baker, et al., 2012, Costello et al., 2015). 3Dp models prepared prior to surgery and used shortly before post-operative care begins in a Paediatric Cardiac Intensive Care Unit (PCICU) can enhance clinical handover between the operating team and the critical care team who are often less familiar with the individual anatomy of the patient. The models are therefore used to demonstrate the pre-operative anatomy of the patient and the procedures performed to the non-operative healthcare professionals. The models could also then be used to anticipate common complications and plan management as required (Laura et al., 2016). Additionally, having an improved understanding of the underlying abnormal anatomy and the procedures undergone by the patients, healthcare providers would be in a more favourable position to provide care (John et al., 2014).

Conventional hand-over proceedings are an efficient model in communicating information and can include succinct presentation of important intraoperative findings, procedures performed and a brief discussion of the required support in intensive care. Such proceedings are particularly effective if performed between providers of the same specialty at each location of care, for example in the operating theatre and PCICU, but they are not necessarily well-equipped to convey broader surgical concepts and clinically relevant anatomical details. Furthermore, in view of the multidisciplinary nature of hand-overs, the use of 3Dp models is especially important since it can improve communication and can enhance interdisciplinary teamwork when faced with differing perspectives and prior knowledge and expertise of the various healthcare providers involved (Laura et al., 2016).

### Summary: 3D-printing in postgraduate surgical training

Although 3Dp models have been used within various training approaches, they are not without limitations. Firstly, despite advancements in technology, it is still not possible to replicate very small structures, and even if it were, it is not always practical to include every structure in the region of interest. For example, in the case of cerebral aneurysm clipping training, blood vessels smaller than one millimeter cannot be produced, and cranial nerves are excluded in the model ( Ryan et al., 2016). Secondly, although it is possible to utilise a combination of materials in varying proportions to mimic real-life tissue, 3Dp models do not reproduce tissue realism to the same extent as cadaveric specimens and therefore do not provide the equivalent tactile experience ( Javan et al., 2016; Jehad et al., 2016). However, surgeons still describe 3Dp models as useful training tools, especially when utilised for learners who are early in their surgical careers ( Ryan et al., 2016). With future advancements in technology and increasing wider popularity, it is perhaps reasonable to expect fidelity to be improved, and costs and production times to be further reduced ( Kook Jin et al., 2015; Ryan et al., 2016) to the extent that 3Dp models will become essential resources in the context of postgraduate surgical training.

## Medical Usage

### Uses of 3D-printing in medical practice

3Dp technology has been used in medicine since before the turn of the century ( Dawood, et al., 2015; Kim et al., 2016), where patients with conditions such as cleft palate could receive patient-specific implants ( Dawood et al., 2015). With development and innovation of the technology driving down costs, the use of 3Dp has become more widespread, eventually resulting in United States Food and Drug Administration approval for several 3Dp devices ( Lee, 2016). As described above, 3Dp has many advantages for surgical usage and has become particularly popular in orthopaedic, maxillofacial, cranial and spinal surgery ( Guenette et al., 2016; Liew et al., 2015; Onerci Altunay et al., 2016; Schwam et al., 2016; Tack et al., 2016; Wiedermann, et al., 2017; Xiao et al., 2016; Yang et al., 2016), whilst a growing number of case studies have described its use in fields such as interventional radiology ( Ghisiawan, et al., 2017; Hossien et al., 2016; Kurup et al., 2015; Schmauss et al., 2015; Shi, et al, 2015; Sodian et al., 2009), paediatric surgery ( Morrison et al., 2015; Schmauss et al., 2015) and oncological surgery ( Al Jabbari et al., 2016; Bernhard et al., 2016; Matsumoto et al., 2016). To date, 3Dp models have been used primarily as surgical guides and for pre-operative surgical planning ( Tack et al., 2016). Using them in complex surgical cases appears to have several benefits: a reduced operative time ( Ghisiawan et al., 2017; Govsa, et al., 2017; Tack et al., 2016; Yang et al., 2016), reduced intraoperative blood loss ( Govsa et al., 2017; Tack et al., 2016; Yang et al., 2016), reduced contrast and radiation exposure ( Ghisiawan et al., 2017; Tack et al., 2016) and better patient education ( Bernhard et al., 2016; Fariha et al., 2014; Govsa et al., 2017; Liew et al., 2015; Yang et al., 2016). Other described benefits include the ability to use 3Dp as an intraoperative guide ( Matsumoto et al., 2016; Wiedermann et al., 2017), to test a surgical approach ( Ghisiawan et al., 2017; Guenette et al., 2016), to visualize minute lesions difficult to appreciate on 2D or 3D images ( Guenette et al., 2016) and as described above, to enhance multi-disciplinary team discussions ( Matsumoto et al., 2016).

Oncological surgery across a range of specialties provide an exemplar demonstration of the potential uses of 3Dp in pre-operative planning. In the case of spinal surgery, CT/MRI visible, MRI compatible 3Dp section of the cervical spine of the patient was designed to test different approaches for the cryoablation of a tumor ( Guenette et al., 2016). The approach was revised, as the model demonstrated an element of spinal cord deformity that was previously not noticed. Additionally, 3Dp models can assist in reducing complications; as tumors commonly encroach upon surrounding structures. Using a 3Dp model as a navigational guide can allow surgeons to proceed more safely. A safe resection margin during an en-bloc cervical spondylectomy for a primary malignant bone tumor has been planned, identifying which neurovascular structures must be sacrificed or could be avoided ( Xiao et al., 2016). In some cases, the pre-operative identification of tumor borders resulted in a smaller incision and less invasive approach, factors that could impact upon recovery ( Al Jabbari et al., 2016; Matsumoto et al., 2016).

One of the more revolutionary uses of 3Dp models involves the creation and use of implants that are specific to the patient’s pathology. For soft-tissue replacement, a model of the lesion can be printed and used as a mould for further processing ( Chung et al., 2014; He et al., 2014). This has been used to successfully close nasal septal perforations ( Onerci Altunay et al., 2016), with an improved retention rate of the implant. Printing a life-size 3Dp model may simply be used to test the best-fitting implant available, reducing operative time and saving on costs. It is a common site to see a ‘wasted’ implant when the surgeon decides upon using a different sized implant. The advantages of adding this extra step have been reported for complex orthopaedic hip replacements ( Tack et al., 2016) and occlusion of congenital septal defects ( Onerci Altunay et al., 2016). Occasionally the 3Dp model may demonstrate an incompatibility with pre-existing implants. This is most common in the case of reconstructive surgery, where a one-size-fits-all approach is not suitable. For example, a 3Dp model of the skull could be delivered to a company for them to produce a custom-made titanium implant ( Tack et al., 2016). The importance of 3Dp models has also been highlighted by interventional radiologists. When repairing lesions in the ascending aorta, testing the model allows the clinician to determine whether or not the supra-aortic vessels would be occluded by a patch or stent. Upon seeing the result, additional steps could be added to the operation to perforate specific areas of the implant ( Ghisiawan et al., 2017) and custom-made devices could be ordered to access the lesion more easily ( Sodian et al., 2009).

Finally, the introduction of biomaterials printing will diversify the uses of 3D printers. Already, biocompatible materials have been used to astounding success. Bronchial splints have been printed from polycaprolactone and surgically attached to the bronchi of children with tracheobronchomalacia ( Morrison et al., 2015). The splints were strong enough to keep the airways open and incorporated a widening mechanism to accommodate airway maturation and growth. In the future, living cells could be incorporated into these 3Dp structures and may eventually solve one of the greatest problems of transplantation medicine, i.e. donor shortage. Taking a major step towards producing human visceral analogues, animal bone, cartilage and skeletal muscle have been printed, while achieving vascularisation of synthetic tissues ( Kang et al., 2016).

### Summary: Medical usage

To date, 3Dp technology is not yet widely incorporated within medicine. The regular printing of models for pre-operative analysis or implant customisation is expensive and only suitable for elective cases. As recommended through describing experiences of regular use ( Schmauss et al., 2015), 3Dp should be reserved for complex cases and clinicians should be responsible for decision making. Eventually, hospitals may begin regularly producing their own equipment for clinical use such as 3Dp surgical retractors ( Lee, 2016), ophthalmology tools ( Hong, 2015) and even simple intravenous bag hooks ( Dawood et al., 2015). The use of high-quality 3Dp models within multidisciplinary team meetings in order to enhance communication may also become established.

## Medical Research

3Dp has become an effective method for producing various devices across a range of specialty fields, including bioengineering, pharmacology, medical instrument production, forensic science and medical science ( Gross et al., 2014; Ventola, 2014). 3Dp devices are useful for point-of-care diagnostics as they have been shown to be able to monitor glucose and lactate ( Gowers et al., 2015), and meter and lyse clinical urine samples for downstream nucleic acid amplification ( Jue et al., 2016). In the pharmaceutical industry, 3Dp in-vitro models can be used to generate multiple pharmacokinetic profiles simultaneously which are then used to develop a prediction model for the pharmacokinetic properties of particular drugs ( Lockwood et al., 2016).

Tissue engineering, which is the practice of combining biomaterials and stem cells, is used to restore damaged tissues and organs. Due to increasing demand, research to improve regenerative medicine technology is needed and to that end, polymer-based scaffolds on which cells grow to produce a matrix must be manufactured ( Steffens et al., 2013). There are several methods to do so but 3Dp allows detailed control of several aspects such as pore geometry, size, interconnectivity and spatial distribution within the scaffold ( Gloria et al., 2012; Park et al., 2012). One study used poly ε-caprolactone (PCL) as the main material to evaluate stem cell interaction with the scaffold. Although there was a significantly lower number of viable cells in the 3Dp scaffolds compared to a control group of cells seeded onto culture plates, it was still possible to observe formation of cell colonies and their attachment to the fibres of the polymer, forming extracellular matrix. This finding demonstrated that the stem cells were indeed interacting with the biomaterial and with each other ( Steffens et al., 2013). Additionally, due to the hydrophobic nature of PCL, such 3Dp scaffolds can take up to three years to degrade, hence rendering them potentially suitable for use in regenerating large defects ( Park et al., 2012).

The development of surgical instruments is following suit in this new era of rapid advances in medical technology. To effectively produce new instruments which confer certain benefits over their classical counterparts, e.g. greater ergonomic user-friendliness, it is necessary to have a rapid workflow from design through to prototyping ( Yamamoto et al., 2015). Using 3Dp technology, instrumental designs can be conceptualised, especially since it is possible to manufacture individual parts of a complex mechanism then assemble them to generate the final product. Prototypes of fully functional medical instruments can be produced if the appropriate materials are used ( Chua, Leong, & Lim, 2010; Kucklick, 2012). Even cases where it is not possible or practical to do so, 3Dp instruments can still support the concept for instrumental design ( Yamamoto et al., 2015).

## Conclusion

Research comparing the educational value of 3Dp with established and traditional methods of anatomy education has been identified and findings support the use of this technology in certain contexts, while primarily financial barriers to the use of 3Dp models in undergraduate anatomy education and postgraduate surgical training have also been identified. While 3Dp can be utilised to supplement existing educational approaches, there are economicand practical implications of introducing this technology that must be considered, in addition to the theoretical basis and evidence supporting the use of 3Dp in education. The range of effective uses for 3Dp in medical practice and research in addtion to medical education and training have also been highlighted. This narrative review provides a preliminary investigation of the current literature, which can provide the basis for a future systematic review and meta-analysis of the use of 3Dp in medical education. As further primary studies are conducted to investigate the value of 3Dp in medical education, a clearer picture of the quality of the evidence supporting 3Dp usage is likely to emerge.

## Take Home Messages

• 3D printing is emerging as an important supplementary learning and teaching resource in undergraduate anatomy education and postgraduate surgical training.
• There are notable applications of 3D printing techology currently used in surgical practice and in medical research.

## Notes On Contributors

Christien Li Ka Hou is a thirdyear medical student at Newcastle University and a researcher at the department of Medicine and Therapeutics, Chinese University of Hong Kong. Currently, his research interests include clinical cardiac electrophysiology, the identification of non-interventional cardiac risk-stratification markers and the use of 3D printing in medicine.

Christopher Kui is a third year medical student at Newcastle University and has previously conducted research with the Epithelial Cell Biology Research Centre at the Chinese University of Hong Kong. He currently enjoys teaching first aid, anatomy and neurology and is interested in the applications of 3D printing in medicine.

Elgin Kah Meng Lee is a third year medical student at Newcastle University and is currently on the committee of the university’s Medical Education society. He has special interests in research towards improving the delivery of medical education and the technology associated with it.

Ho Cheuk Sang received his pharmacy degree from the Chinese University of Hong Kong in 2012, and is currently a third year medical student at the same university. His research interests include innovative teaching methods in undergraduate medical education and the use of 3D printing in medicine.

Dr Sunny H. Wong is an Assistant Professor at the Department of Medicine and Therapeutics in the Chinese University of Hong Kong. Specializing in digestive diseases, his research interest is to translate scientific discoveries into clinical applications for diagnosing or treating gastrointestinal diseases. He has published over 50 peer-reviewed articles including papers in *Nature Genetics*, *Nature Communications* and *Gut.*

Dr William K.K. Wu is an Assistant Professor in the Department of Anaesthesia and Intensive Care at the Chinese University of Hong Kong. His research interests include cellular signaling and genomics. He has published over 180 peer-reviewed articles, including papers in *Cell Research, Nature Communications* and *Gut.*

Dr. Wing Tak Jack Wong earned his Ph.D. in Physiology from the Chinese University of Hong Kong in 2009. Dr. Wong was awarded a postdoctoral fellowship from American Heart Association at Stanford University before becoming an Assistant Professor of Houston Methodist Research Institute and Weill Cornell Medicine of Cornell University in 2013. He has published over 60 research papers and review articles in the area of vascular medicine and biology in high impact journals including *Circulation*, *Circulation Research*, *Cell Metabolism*, *Hypertension*, *Diabetes*, *Antioxidants & Redox Signaling*, *Arteriosclerosis*, *Thrombosis and Vascular Biology*, *Cardiovascular Research*, *Journal of Hypertension*, and *Radiology* (Citation over 2000 with a h-index of 28).

Miss Jessika Voll is a specialist registrar in General Surgery rotation at Health Education North East. After graduating medicine from University of Tartu, Estonia in 2008, her research interest concerns novel ways of teaching and training for medical students and junior doctors. She also has Masters in Medical Education from University of Nottingham, UK 2015.

Professor Guangping Li is an academic cardiologist, and currently serves as Chief and Professor of Cardiology at the Second Hospital of Tianjin Medical University. He leads a productive research group and has contributed to more than 200 publications in leading cardiology journals.

Dr. Tong Liu graduated from Tianjin Medical University in 2000, and achieved his PhD in 2006. He received training in the research of electrophysiology in the Heart Institute, Cedars-Sinai Medical Center, Los Angeles, from 2009 to 2010. He has particular interests in cardiac electrophysiology, and for the past decade he has focussed on research involving atrial fibrillation. He is the Youth Committee Member of the Chinese Society of Pacing and Electrophysiology (CSPE) and published over 100 articles in peer reviewed medical journals.

Bryan Yan is an interventional cardiologist and Associate Professor in the Department of Medicine and Therapeutics, Faculty of Medicine, The Chinese University of Hong Kong and Adjunct Associate Professor at the Department of Epidemiology and Preventive Medicine, Monash University, Melbourne, Australia. Prof. Yan graduated from the University of Melbourne and received his training in cardiology and interventional cardiology at the Royal Melbourne Hospital, Australia. He subsequently underwent a clinical fellowship year in vascular medicine and peripheral vascular interventions at the Massachusetts General Hospital and research fellowship at the Harvard Medical School in Boston, United States.

Jessica Chan is a researcher at Department of Education, University of Oxford. Her research interests include teacher education and professional development, assessment, pedagogy and cultural-historical activity theory.

Dr. Gary Tse is an Assistant Professor at the Department of Medicine and Therapeutics and a Principal Investigator at the Li Ka Shing Institute of Health Sciences of the Faculty of Medicine, The Chinese University of Hong Kong. Dr. Tse has published more than 54 full publications in international journals such as *Stroke*, *Heart Rhythm*, *Europace*, J *ournal of the American Heart Association* and *Frontiers in Physiology.*

Dr Iain D. Keenan is a Lecturer in Anatomy and plays key roles in the management and delivery of the medical degree programme within the School of Medical Education at Newcastle University. In addtion to a PhD in Biology from the University of York, he holds a Masters in Medical Education from Newcastle University and his current research interests concern the development and evaluation of innovative, creative and digital learning approaches in anatomy education. His postdoctoral research at the University of Dundee, the University of St Andrews and Newcastle University involved investigating the molecular regulation of gene expression in vertebrate growth and development. He has authored several articles in leading anatomy education and developmental biology journals.

## Declarations

Dr Iain Keenan declares a conflict of interest as current theme guest co-editor of “Life Sciences in an Integrated Curriculum” in MedEdPublish. There are no other conflicts of interest to declare.
