# Supplementary material for: The role of 3D printing in anatomy education and surgical training: A narrative review
Source: MedEdPublish (2016). 2017 Jun 6;6:92. [Version 1] doi: 10.15694/mep.2017.000092 (PMC10885259; doi:10.15694/mep.2017.000092)
Supplement: Peer review history

# Reviewer report: Reviewer response for version 1

**DOI:** 10.21956/mep.19252.r27950

**Published:** 2017-06-18

**Authors:** Michael SH Wan

**Affiliations:** 1 University of Notre Dame

This review has been migrated. The reviewer awarded 4 stars out of 5 A well written review of the use of 3Dp in medical education including the use in post graduate training. We used to have dissection, prosected specimens in the lab which involved significant amount of setup and maintenance. With the advance in 3D printing technology and image capturing software, the cost of set up and the subsequent production of each model will definitively be getting more affordable. With this review, schools who have interest in exploring this option could be motivated to pilot the use of 3Dp models in teaching anatomy. The use of pathology specimens from the museum to teach the students are limited by the availability and conditions of the specimen, 3Dp could easily supplement this. A question would be how well the 3D models (usually quite rigid and hard) will be compared to the real prosected specimen where students could move away arteries, tendons or other structure to expose the deeper region of study? The feel of the intestines and the brain would be very different with a plastic printed model. Will there be any systems or structures that would still required the old style classic dissection/prosected specimen for teaching?Further pilot study on the outcomes (e.g. assessment performance) comparison between classical and 3Dp teaching would be useful.

Reviewer Expertise:

NA

No decision status is available

# Reviewer report: Reviewer response for version 1

**DOI:** 10.21956/mep.19252.r27949

**Published:** 2017-06-15

**Authors:** Stuart Irving

**Affiliations:** 1 University of East Anglia

This review has been migrated. The reviewer awarded 4 stars out of 5 This paper reviews the current and future position of 3d printed anatomical models in the undergraduate, postgraduate and therapeutic environments. The review clearly sets out to describe this technology as a supplement modality to existing teaching aids. The review is very comprehensive and highlights novel areas for the production of non-cadaveric aids such as anatomical variation that are not commonly seen and are not available “to order” in cadaveric material. The costs and time taken to produce are discussed in depth as is their suitability for home use which is not available for cadaveric or plastinated material. I would have liked to have seen a little more detail in relation to the cost effective production of postgraduate training aids and the advances made in terms of different 3d printing materials in terms of colour or texture that could give an idea of where we may be with this exciting technology in the near future.

Reviewer Expertise:

NA

No decision status is available

# Reviewer report: Reviewer response for version 1

**DOI:** 10.21956/mep.19252.r27948

**Published:** 2017-06-13

**Authors:** Trevor Gibbs

**Affiliations:** 1 AMEE

This review has been migrated. The reviewer awarded 3 stars out of 5 An interesting paper to read and good to see that involvement of students. There were a lot of authors and it would have been useful to see how each contributed to the paper given their background and interests. In such lost authors must I believe always take into account reviewers feelings regarding this large number, given that in many instances co-authors appear on the paper as organisational additives rather than constructive co-authors- i would hope in this case that the opposite occurs here.I believe that this paper is worth reading and as the authors admit in their conclusion that this paper "provides a preliminary investigation of the current literature, which can provide the basis for a future systematic review and meta-analysis of the use of 3Dp in medical education." There is still much work to be done in this area but the search undertaken has provided a substantial amount of important literature for others to move forward with their research.To be constructively critical :I would have liked the search to be conducted over a wider base- I feel that there is a lot of grey literature around this subjectI wonder why only PubMed was searched- given anatomy as a basic science then I do think a search involving other data bases would / could have been more productiveIt is important to look at the search methodology, which the authors have begun to describe, but issues such as number of papers found, inclusion / exclusion criteria, papers discarded, final number etc. are important.Given that it is a narrative review, I worried that the search was too wide and would have preferred to read a review regarding specifically undergraduate education.I feel that this is probably supported by the sometimes mixed batch of outcomes in each of the chapter. The purpose of any review is to come to major conclusions and given the wider net that was cast here, the conclusions appeared somewhat superficialHowever, given these points, I still feel that the paper idea useful read to those involved in the subject

Reviewer Expertise:

NA

No decision status is available

# Reviewer report: Reviewer response for version 1

**DOI:** 10.21956/mep.19252.r27947

**Published:** 2017-06-13

**Authors:** Ken Masters

**Affiliations:** 1 Sultan Qaboos University

This review has been migrated. The reviewer awarded 5 stars out of 5 A very useful paper, identifying the value of using 3-D printing (3Dp) in anatomy education and surgical training. The paper adequately address the current potential and limitations of 3Dp in these fields, and will serve as a useful introduction to the topic for any medical school.It does, however, have a few minor issues that could be addressed.• The penultimate paragraph in the Introduction could be re-structured. The previous paragraph gives an overview of different methods of anatomy teaching. Then this paragraph introduces the topic of 3Dp. And then it returns to a conclusion of the previous paragraph with “The optimal method of teaching anatomy is still widely debated….”• The Introduction is rather thin on the background to surgical training, with the focus on anatomy education. There is a single sentence (“The importance of anatomy also extends to those in surgical specialties due to the direct relevance of the discipline to clinical practice.”) and then all other connections to surgical training would have to be read into the text by the reader. Indeed, the detailed and valuable discussion in the paper of the value of 3Dp to surgical training indicates that the authors have done themselves a disservice by not addressing it more directly in the Introduction.• If the search was designed to find all instances of 3Dp in medical education, then setting the start date to 1965 is rather excessive. 3Dp began in the 1980s, with the first patents issued from the mid-1980s. Even in the 1990s, there was very little application of the technology outside very expensive industrial usage, so any articles earlier than that would merely be noise in such a search. This is supported by the publication dates of the sources cited. So, a quick scan of any (even a Wikipedia) article on the topic would have allowed the search to be more focussed.• Similarly, the authors should state clearly (and I am assuming this to be the case), that the search was a simple “All text” search of Medline, rather than searches on keywords and/or other databases accessed by PubMed.• I would have liked the methodology to show a little more detail of the application of the exclusion and inclusion criteria (in a diagram, if possible) as is frequently performed in such a search.In spite of these criticisms, the essence of the paper (the information drawn from the literature) is well presented, and extremely valuable. The rather tentative nature of the conclusions by the authors is primarily because of the novelty of the technology – I am happy to see such a paper at a relative early stage of the technology’s development so that others may have some guidance on the topic, rather than waiting until all the technological and other issues have been addressed, but at the cost of numerous and expensive explorations in the dark. It will be interesting to revisit this topic in 5 or 10 years’ time to see what has happened in the field.

Reviewer Expertise:

NA

No decision status is available
